# Injectable dual-affinity self-assembling peptide hydrogels for local controlled delivery of therapeutic antibodies

**DOI:** 10.1016/j.ajps.2026.101189

**Published:** 2026-08-12

**Authors:** Weiping Cui, Limin Zhang, Chang Yang, Bing He, Hua Zhang, Xueqing Wang, Zhiwen Zhang, Qiang Zhang, Wenbing Dai

**Affiliations:** a Beijing Key Laboratory of Advanced Pharmaceutical Preparation, State Key Laboratory of Natural and Biomimetic Drugs, School of Pharmaceutical Sciences, Peking University, Beijing 100191, China; b School of Pharmacy, Key laboratory of smart drug delivery (Ministry of Education) & National key laboratory of complex drug formulations for overcoming delivery barriers, Fudan University, Shanghai 201203, China

**Keywords:** Peptide hydrogel, Dual-affinity hydrogel, Co-assembled peptide, Local antibody delivery, Trastuzumab, Collagen type I-binding peptide

## Abstract

Therapeutic monoclonal antibodies have revolutionized the treatment of cancers, infectious diseases and immune disorders; however, their efficacy is compromised by limitations associated with systemic administration, including systemic toxicity, poor patient compliance and inadequate drug concentration at pathological sites. Hydrogels are promising carriers for localized antibody delivery, but conventional formulations fail to simultaneously address the dual challenges of uncontrolled antibody release and insufficient hydrogel retention at the target site. Herein, leveraging a peptide co-assembly strategy, we engineered an injectable dual-affinity self-assembling peptide hydrogel for localized antibody delivery. This hydrogel integrates three components: peptide FEK as the hydrogel matrix, MEP-FEK incorporating 4-mercaptoethylpyridine (MEP) for non-covalent antibody binding, and C_1_BP-FEK functionalized with the collagen-binding peptide TKKTLRT for tumor extracellular matrix anchoring. The dual-affinity hydrogel enabled controlled antibody release, exhibiting only 40% cumulative release by Day 90 and an 8.8-fold reduction in initial diffusion rate. In proof-of-concept studies using SKOV3 tumor-bearing mice, the hydrogel carrying trastuzumab as a model therapeutic antibody enhanced local antibody retention, reduced. systemic exposure, exhibited negligible toxicity, and achieved a tumor inhibition rate of 68% This dual-affinity strategy overcomes limitations of conventional affinity-based hydrogels, providing a biocompatible and versatile platform for localized antibody therapy in oncology and other biomedical applications.

## Graphical abstract

Conventional antibody-loaded hydrogels suffer from uncontrolled release, intricate antibody modification and poor local retention. We developed an injectable dual-affinity peptide hydrogel via co-assembly of FEK (hydrogel matrix), MEP-FEK (antibody-binding), and C₁BP-FEK (collagen-anchoring) peptides, enabling controlled antibody release, robust tumor retention and enhanced local antitumor efficacy with minimal systemic toxicity.

**Figure:**
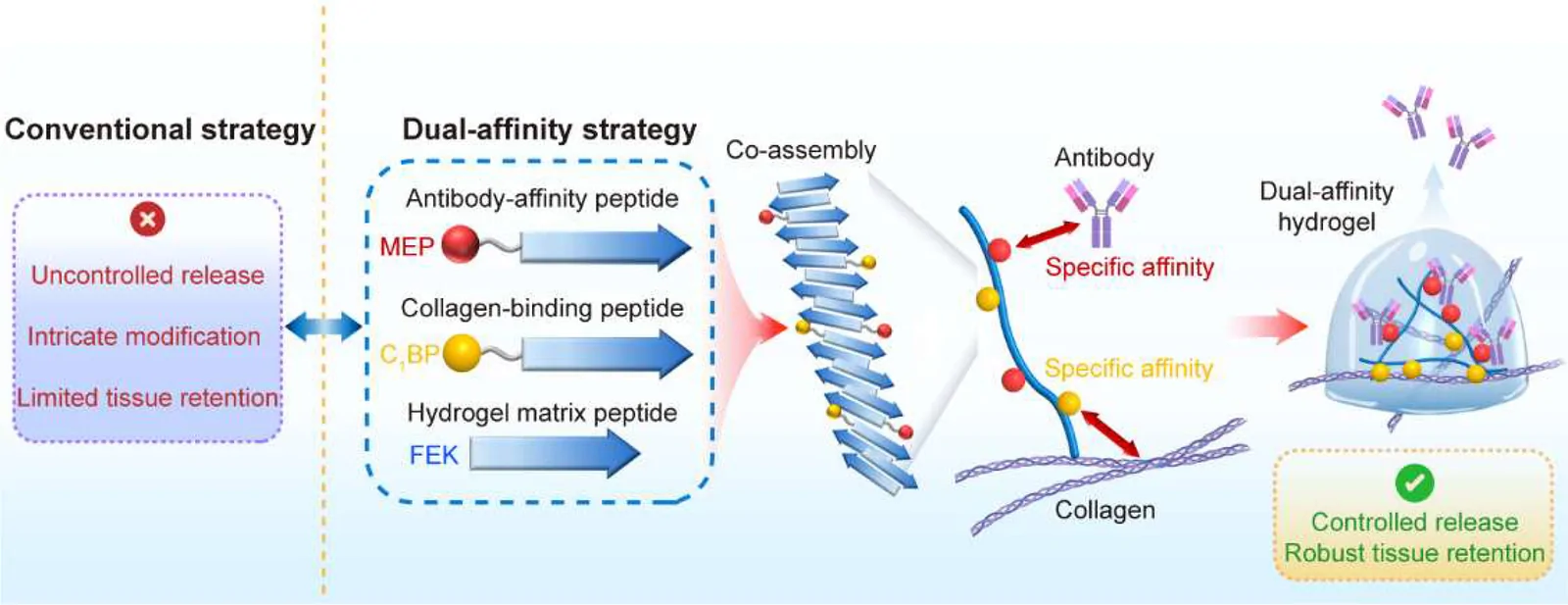

## 1 Introduction

Monoclonal antibodies (mAbs) have revolutionized the treatment of malignancies, infectious diseases and autoimmune diseases due to their remarkable therapeutic efficacy [1,2]. However, clinically available mAbs suffer from inherent limitations, including systemic adverse effects associated with high-dose systemic administration, poor patient compliance resulting from frequent dosing regimens, and inadequate antibody concentration at pathological sites [[3], [4], [5], [6]]. For instance, intravenous or subcutaneous mAb delivery shows extremely low *in vivo* efficiency, with < 0.01% of the injected dose accumulating per gram of solid tumor tissue [7].

Local delivery systems for mAbs, such as intratumoral or intraarticular administration, enable high-efficiency drug enrichment at target sites while minimizing systemic distribution, thereby reducing dose-related toxicities. This approach holds pivotal importance in malignant tumor therapy and has been validated by clinical studies [4,8]. Hydrogels have emerged as promising local delivery platforms and ideal reservoirs for protein therapeutics, with great potential for optimizing drug release profiles mainly via diffusion [[9], [10], [11]]. However, mesh sizes of most hydrogels far exceed the hydrodynamic diameters of proteins, which hinders drug retention in the matrix and leads to rapid, uncontrollable antibody release [12,13]. Although covalent conjugation-based hydrogels can mitigate this issue, existing systems necessitate intricate modification of the antibody, which not only increases operational complexity but also risks compromising antibody bioactivity and restricts compatibility with a broad spectrum of antibodies [14,15]. In contrast, the non-covalent binding affinity strategy, which exhibits superior efficacy in immobilizing mAbs on local carriers, offers robust mAb anchoring and eliminates the need for chemical modification of the antibody, thereby preserving its bioactivity [16,17]. Beyond the challenge of precise controlled drug release, another critical yet frequently overlooked limitation lies in the rapid local clearance of hydrogel carriers. The tumor microenvironment exhibits elevated interstitial fluid pressure and vascular hyperpermeability, which readily induce displacement or degradation of hydrogels, severely limiting their *in situ* retention [18]. This renders the extracellular matrix (ECM)-affinity strategy one of the most effective approaches for enhancing sustained *in situ* retention [19,20]. Thus, developing an uncomplicated and universally compatible localized delivery system that avoids antibody modification, achieves sustained release, and prolongs local retention holds significant clinical relevance for improving the therapeutic efficacy of mAb therapy and patient compliance.

Among the various hydrogel platforms, self-assembling peptide hydrogels have emerged as ideal reservoirs for macromolecular drugs due to their biocompatibility, biodegradability, high water content, and capacity to encapsulate therapeutic proteins while preserving their native conformations [[21], [22], [23]]. Herein, we propose a dual-affinity strategy leveraging the co-assembly of antibody- and tumor ECM-affinity self-assembling peptides for sustained local mAb delivery. To achieve the desired dual functions—antibody binding and ECM anchoring—we selected two well-characterized functional moieties: 4-mercaptoethylpyridine (MEP) and a peptide motif targeting type I collagen. MEP, a small-molecule antibody affinity ligand, binds to the conserved Fc/Fab domains of IgG via hydrophobic interactions (Kd = 1.16 × 10^–7^ M) [24]. Endowed with good aqueous solubility, facile synthesis, and low cost, MEP is a versatile ligand for antibody purification. For ECM anchoring, type I collagen, which is overexpressed in the tumor interstitial ECM and is a key component of tumor desmoplasia [25], was targeted using the TKKTLRT motif, a specific collagen-binding sequence [26,27].

**Scheme 1:**
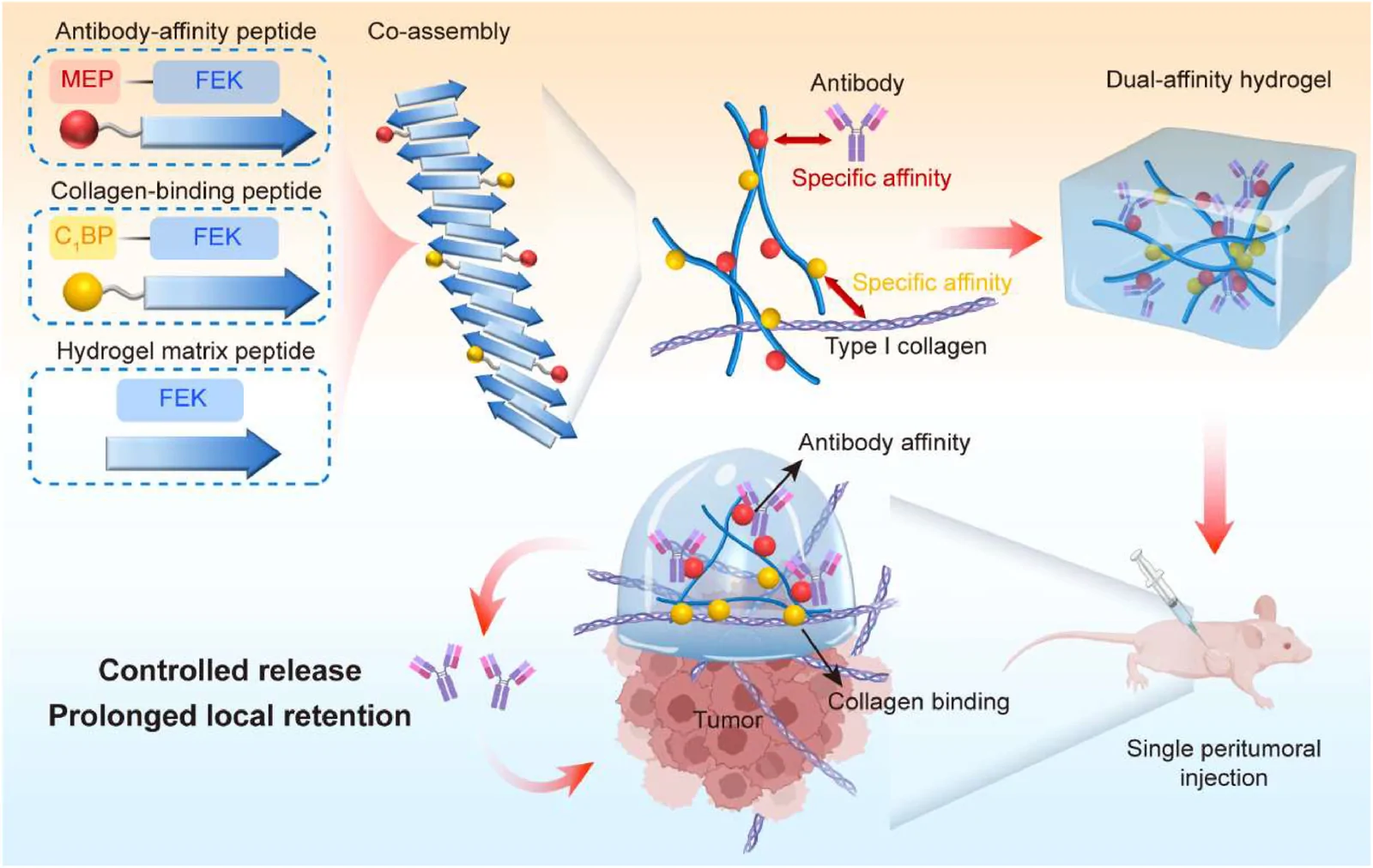
Schematic representation of the dual-affinity self-assembling peptide hydrogel for local antibody delivery. The injectable dual-affinity hydrogel was fabricated via the co-assembly of three functional peptides: the ionic-complementary FEK (hydrogel matrix), MEP-FEK (antibody-affinity peptide with non-covalent antibody binding capability), and C₁BP-FEK (collagen-binding peptide targeting tumor ECM type I collagen). Upon peritumoral injection, the dual-affinity design enables sustained antibody release and attenuated burst release through MEP-mediated antibody binding and prolonged local retention via C_1_BP-mediated collagen anchoring, thereby enhancing local antitumor efficacy while minimizing systemic exposure.

We used the ionic-complementary self-assembling peptide FEFEFKFK (abbreviated as FEK) as the hydrogel matrix, together with two functional peptides: MEP-FEK (for non-covalent antibody-affinity) and C_1_BP-FEK (for tumor ECM anchoring) (Scheme 1). This design was aimed at simultaneously retarding mAb release and prolonging the co-retention of hydrogel and antibody at the tumor site, thereby boosting local antitumor efficacy [[26], [27], [28]].

Furthermore, as a proof of concept, we employed the clinically approved anti-HER2 monoclonal antibody trastuzumab (Tra) as a model drug to fabricate drug-loaded dual-affinity hydrogels. Following comprehensive assessment of its mechanical properties and binding affinity via pull-down and enzyme-linked immunosorbent assays, the co-assembled hydrogel was optimized at a molar ratio of 1:1:8 (MEP-FEK:C_1_BP-FEK:FEK). *In vitro* and *in vivo* studies confirmed that this dual-affinity hydrogel significantly sustained mAb release, enhanced tumor local retention, and exhibited prolonged and potent antitumor efficacy and minimal systemic toxicity. This dual-affinity hydrogel, constructed via a peptide co-assembly framework integrating two functional motifs, offers distinct advantages: (1) preserved antibody integrity and activity, (2) attenuated burst release and sustained mAb release, and (3) enhanced hydrogel-antibody co-retention, minimizing systemic exposure and improving therapeutic efficacy. This versatile localized antibody delivery platform provides a promising avenue for enhancing the efficacy and safety profiles of antibody-based therapeutic regimens.

## 2 Materials and methods

### 2.1 Materials

Rink Amide-MBHA Resin (loading capacity: 0.25 mmol/g) and all 9-fluorenylmethoxycarbonyl (Fmoc)-protected amino acids were obtained from GL Biochem (Shanghai) Ltd. (Shanghai, China). Tra (Herceptin®) was acquired from Roche Pharmaceutical Co., Ltd. (Shanghai, China). Fluorescein 5-isothiocyanate (FITC)-labeled human immunoglobulin G, rat tail tendon collagen type I, rabbit primary antibody against type I collagen, cyanine 3 (Cy3)-conjugated goat anti-rabbit IgG secondary antibody, methyl thiazolyl tetrazolium (MTT), Hoechst 33342, and Lyso-Tracker Green were purchased from Beijing Solarbio Science & Technology Co., Ltd. (Beijing, China). DAPI staining solution was obtained from Beyotime Biotechnology (Shanghai, China). Hematoxylin-eosin (H&E) staining kit was supplied by Wuhan Servicebio Technology Co., Ltd. (Wuhan, China).

### 2.2 Synthesis of peptide and peptide derivatives

The peptides FEFEFKFK-NH_2_ (FEK) and TKKTLRT-SGSG-FEFEFKFK-NH_2_ (C_1_BP-FEK) were synthesized via solid-phase peptide synthesis following previously reported protocols [29]. For the synthesis and coupling of MEP, 4-vinylpyridine (100 mM) was slowly added to 3-mercaptopropionic acid (100 mM), stirred at 85 °C until solidification, cooled to 50 °C, and then mixed with water under stirring. The solid product was filtered, washed sequentially with water and propanol, and dried to obtain MEP. MEP was coupled to FEK by mixing FEK-conjugated resin, MEP, O-benzotriazole-N,N,N’,N’-tetramethyluronium hexafluorophosphate, and N,N-diisopropylethylamine (DIEA) at a molar ratio of 1:4:4:10 in dimethylformamide, followed by continuous agitation at room temperature overnight in a solid-phase synthesis column, and crude MEP-FEFEFKFK-NH_2_ (MEP-FEK) was obtained using the same cleavage and precipitation protocol as used for FEK and C_1_BP-FEK.

For fluorescent dye conjugation, peptide FEK or MEP-FEK (5 µmol, 5 eq.) was mixed with cyanine 5.5 (Cy5.5) NHS ester or FITC NHS ester (1 µmol, 1 eq.) in anhydrous dimethyl sulfoxide (DMSO), and 2 µL (6 eq.) of DIEA was added. The reaction mixture was stirred in the dark at room temperature for 24 h, yielding Cy5.5-labeled peptides (FEK^Cy5.5^ and C_1_BP-FEK^Cy5.5^) and FITC-labeled peptide (FEK^FITC^).

All peptides and their derivatives were purified via preparative high-performance liquid chromatography (HPLC) following the protocols reported previously [29,30]. The purified products were verified via electrospray ionization-mass spectrometry (ESI-MS) using an AB Sciex API 4000 triple quadrupole mass spectrometer (USA), and their purity was determined via analytical HPLC on a Shimadzu LC-20A system (Japan) equipped with an XBridge® Peptide BEH C_18_ column.

### 2.3 Peptide self-assembly and hydrogel preparation

#### 2.3.1 Peptide self-assembly

Lyophilized peptides were dissolved in phosphate-buffered saline (PBS; 0.01 M, pH 7.4) to 100 µM and incubated at room temperature for 24 h to form nanofibers (NFs).

#### 2.3.2 Peptide co-assembly

Peptide powders were first mixed at the desired molar ratios and dissolved in acetonitrile (ACN)-water mixture to disrupt any pre‑formed self‑assemblies. The solutions were then lyophilized to remove the solvent. To determine the optimal ACN concentration, a series of ACN‑water mixtures with gradient ACN proportions were prepared, maintaining a final peptide concentration of 5 mM. The resulting assembly states were characterized via thioflavin T (ThT) assay and transmission electron microscopy (TEM). After optimizing the ACN ratio, all peptide powders were dissolved in the optimal ACN-water solution, lyophilized, reconstituted in Milli-Q water, vortexed for 10 min, and the pH was adjusted to 7.0 to induce co-assembly into NFs or hydrogels.

#### 2.3.3 Hydrogel preparation

Peptides were mixed at pre-optimized molar ratios as follows: dual-affinity hydrogel (Dual-affinity Gel) was formulated at MEP-FEK:C_1_BP-FEK:FEK molar ratio of 1:1:8; antibody-affinity hydrogel (MEP-Gel) was formulated at MEP-FEK:FEK molar ratio of 1:9; and collagen-affinity hydrogel (CBP-Gel) was formulated at C_1_BP-FEK:FEK molar ratio of 1:9; the control hydrogel (Gel) consisted of FEK only. The peptide mixtures were dispersed in Milli-Q water, dissolved in 40% ACN aqueous solution, lyophilized, and reconstituted in Milli-Q water to 5 mM. The pH was adjusted to 7.0 with 0.2 M NaOH, and the mixture was allowed to stand until hydrogel formation (inverted tube no flow). For drug-loaded hydrogels, Tra or IgG-FITC (2 g/l) was mixed with peptide solution before gelation.

### 2.4 Structural and morphological characterization

#### 2.4.1 TEM

Self-assembled peptide solutions were dropped onto carbon-coated copper grids, stained with 2% uranyl acetate for 3 min. TEM was performed using a JEM-1400 instrument at an acceleration voltage of 100 kV.

#### 2.4.2 Circular dichroism spectroscopy

Peptide solutions (20–100 µM) were placed in a 1 mm quartz cuvette at 20 °C and scanned over a wavelength from 195 to 260 nm at 0.5 nm/s, with a bandwidth of 1 nm and response time of 2 s. Data represent the average of three scans.

#### 2.4.3 Fluorescence spectroscopy

ThT fluorescence was measured using excitation and emission wavelengths of 450 and 482 nm, respectively, for β-sheet detection. For release studies, IgG-FITC fluorescence was detected at excitation and emission wavelengths of 490 and 520 nm, respectively.

#### 2.4.4 Critical aggregation concentration (CAC)

CAC of peptide molecules was determined via the ThT fluorescence assay. ThT exhibits a marked increase in emission fluorescence at 482 nm upon excitation at 450 nm when bound to β-sheet amyloid fibrils [31]. Gradient peptide concentrations (0–500 µM) containing 25 µM ThT were incubated overnight. Fluorescence intensity ratio (I_x_/I_0_) was plotted against peptide concentration. CAC was determined as the intersection of two linear fits of data above and below the inflection point.

### 2.5 Affinity measurements

#### 2.5.1 Peptide-Tra binding

Microscale thermophoresis (MST) measurements were used to evaluate the binding affinity between the antibody-affinity peptide and Tra. First, Tra was labeled with NT-647-NHS using a protein labeling kit. The antibody-affinity peptide MEP-FEK solutions with gradient-diluted concentrations were then mixed with labeled Tra at a 1:1 ratio (v/v). Finally, the capillaries were immersed in the mixtures, filled with solution, and MST (Monolith NT.115) measurements were performed to determine the final binding affinities (*K*_d_).

#### 2.5.2 Peptide-collagen I binding

The ability of collagen-affinity peptide to bind to collagen was assessed *in vitro* using a modified ELISA [26]. Briefly, the collagen-affinity peptide C_1_BP-FEK^FITC^ (0–500 µM) was added to 96-well plates precoated with collagen type I and incubated at 37  °C for 2 h, and then the plates were washed five times to remove the unbound proteins. The fluorescence intensity at 525 nm was recorded using an multi-mode microplate reader (BioTek Synergy Neo2) under excitation light at 495 nm. The dissociation constant (*K*_d_) for the binding of the collagen-affinity peptide to collagen was calculated using GraphPad Prism 8.0.

### 2.6 Co-assembly ratio screening

#### 2.6.1 Single affinity peptide ratio screening

For antibody-affinity peptide, FITC-labeled IgG (IgG^FITC^, 0.276 nmol) was mixed with peptide solutions at different molar ratios (FEK:MEP-FEK = 1:0, 19:1, 9:1, 4:1 and 0:1) to prepare 20 µl peptide hydrogels (5 mM). After incubation for 30 min, samples were diluted to 100 µl with PBS (0.01 M, pH 6.5), washed five times with 1 ml PBS via high-speed centrifugation, and resuspended. The fluorescence intensity was measured to determine the optimal co-assembly ratio.

For collagen affinity peptide, using the same protocol as above, 0.276 nmol FITC-labeled collagen type I was mixed with peptide solutions at molar ratios of FEK:C_1_BP-FEK = 1:0, 95:5, 90:10, 85:15, 80:20, 0:1 to form 20 µl hydrogels (5 mM). Post-incubation, washing, and centrifugation, fluorescence was detected to determine the optimal co-assembly ratio.

#### 2.6.2 Screening for optimal dual-affinity peptide ratio

ELISA was employed to screen for the optimal ternary ratio (MEP-FEK:C_1_BP-FEK:FEK = 10:10:80, 5:5:90, 5:15:80, 15:5:80). Briefly, 96-well plates were coated with collagen type I (4 µg/ml, 100 µl/well) at 4 °C overnight, blocked with 2% bovine serum albumin (BSA) in PBS for 2 h, and then incubated with 100 µl antibody-loaded hydrogels (2 mM, containing 0.276 nmol IgG^FITC^) at 37 °C for 2 h. After five washes with PBS, fluorescence intensity (excitation 495 nm, emission 525 nm) was measured to evaluate antibody retention and determine the optimal ternary ratio.

### 2.7 Rheological characterization and injectability test

Rheological properties of the peptide hydrogels were evaluated using a rheometer. All measurements were performed at 25 °C with a fixed gap distance of 1.0 mm. For frequency sweep tests, the storage modulus (G’) and loss modulus (G’’) were recorded over a frequency range of 0.1–100 rad/s under a constant strain of 1% (within the linear viscoelastic region) to evaluate gel mechanical stability. Thixotropic behavior was assessed via the three-interval thixotropy test (3ITT) to simulate the injection process: the test was conducted in oscillation mode at a fixed frequency of 10 rad/s, with strain sequentially switched from 1% (initial gel state) to 100% (shear-induced disruption) and back to 1% (structure recovery), monitoring the dynamic recovery of G’ and G’’ to verify injectability.

To assess injectability and dosage uniformity, each hydrogel formulation was loaded into a 1 mL syringe (BD, 26 G needle). A volume of 100 µl was manually extruded, collected and weighed. Assuming a density of approximately 1 g/ml for all formulations, the measured mass (mg) directly corresponds to the injection volume (µl). The coefficient of variation (CV) was calculated as (SD/mean) × 100%. The extrusion process was photographed to visually confirm smooth flow and rapid gelation.

### 2.8 Cell culture and animal models

Human ovarian cancer SKOV3 cells were obtained from the Cell Bank of the Chinese Academy of Sciences (Shanghai, China). Cells were cultured in RPMI 1640 medium supplemented with 10% (v/v) heat-inactivated fetal bovine serum (FBS) and 1% (v/v) penicillin-streptomycin at 37 °C in a humidified atmosphere containing 5% CO₂.

BALB/c nude mice (18–20 g, female) were purchased from Vital River Laboratory Animal Center (Beijing, China) and female BALB/c mice (18–20 g, female) were obtained from Peking University Health Science Center (Beijing, China). The animals (4–5 mice per cage) were housed under specific pathogen–free conditions with controlled humidity at ∼25 °C. The photoperiod was set to a 12-h cycle of light and darkness. The animals had unrestricted access to standard food and water. To establish tumor models, a suspension of 100 µl SKOV3 cells (2 × 10^6^) was subcutaneously injected into the right axilla of each mouse. When the tumor volume was approximately 50–100 mm^3^, the mice were used for subsequent experiments, such as fluorescence imaging and antitumor study. All animal experiments were conducted at the Center for Experimental Animals, Peking University, adhering to the guidelines set forth by the National Institutes of Animal Care and Use Committee. The experiments were approved by the Institutional Animal Care and Use Committee of Peking University.

### 2.9 In vivo retention and colocalization

#### 2.9.1 Determination of collagen type I levels in tumor tissue

Tumor tissues were fixed in 4% paraformaldehyde, embedded in paraffin, and sectioned into 5 µm-thick slices. Immunohistochemistry was performed by incubating slices with rabbit anti-collagen I primary antibody at 4 °C overnight, followed by horseradish peroxidase-conjugated goat anti-rabbit secondary antibody and 3,3′-diaminobenzidine chromogen. Nuclei were counterstained with hematoxylin. The sections were dehydrated, mounted, and examined under a bright-field microscope to assess collagen type I content and distribution.

#### 2.9.2 In vivo retention of dual-affinity hydrogels

Two imaging hydrogels were prepared: Cy5.5-labeled hydrogel (FEK^Cy5.5^:FEK = 1:9, 5 mM) and Cy5.5-labeled dual-affinity hydrogel (MEP-FEK:C_1_BP-FEK:FEK^Cy5.5^:FEK = 1:1:1:7, 5 mM). BALB/c mice (*n* = 3) were subcutaneously injected with 100 µl of each formulation into the right flank. At predetermined time points (1, 3, 6, 9, 12, 15 and 20 d post-injection), the mice were anesthetized and imaged using an IVIS SPECTRUM small animal imaging system (PerkinElmer, USA) with excitation at 670 nm and emission at 710 nm.

#### 2.9.3 Colocalization of dual-affinity hydrogels with type I collagen

Tumor-bearing mice were peritumorally injected with 100 µl Cy5.5-labeled control or dual-affinity hydrogel. After 2 d, tumors were excised, embedded in optimal cutting temperature medium, and sectioned into 10 µm slices using a cryostat (Leica CM1950). The sections were baked at 37 °C for 10–20 min, fixed and washed three times with PBS (1×, pH 7.4). They were then stained with rabbit anti-collagen I primary antibody (1:200), Cy3-conjugated goat anti-rabbit secondary antibody (1:500), and DAPI. The sections were visualized using the automated quantitative pathology imaging system (Vectra Polaris).

### 2.10 In vitro release study

The release kinetics of FITC-labeled IgG from peptide hydrogels were assessed by monitoring the cumulative fluorescence in the release medium over 90 d 200 µl IgG^FITC^-loaded peptide hydrogels (containing 400 µg FITC-labeled IgG), specifically IgG@Dual-affinity Gel, IgG@MEP-Gel and IgG@Gel, was injected into the bottom of individual 500 µl centrifuge tubes and incubated overnight at 37 °C. Thereafter, 200 µL of PBS (1×, pH = 6.5) containing 0.1% BSA, 0.024% NaN_3_ was added as the release medium. The samples were incubated at a constant temperature at 37 °C. At predetermined intervals, 100 µl of the release medium was collected for analysis using a Shimadzu RF-6000 Spectro fluorophotometer (excitation = 490 nm; emission = 520 nm). Fresh aliquots of release medium were subsequently added to restore the system to its initial volume.

$$M_{t}=\sum C_{i}\times V_{0}+C_{t}\times V$$

Where *M_t_* represents the cumulative release amount at time *t; C_t_* denotes the drug concentration in the medium at time *t; V_0_* is the volume of the sample taken during each measurement, specifically 100 µl; and *V* refers to the total volume of the release medium, which was 200 µl. The apparent diffusivity (D_app_) of IgG within peptide hydrogels was estimated by fitting the initial release data to the non-steady state Fickian diffusion equation, which correlates M_t_/M_∞_ with t^0.5^ [17].

### 2.11 Fluorescent labeling of Tra

Tra was conjugated with fluorescent dye Cy5.5 activated ester (Cy5.5 NHS ester) in an alkaline environment. Tra was dissolved in a 1 mol/l Na₂HPO₄ solution, and Cy5.5 NHS ester (dissolved in DMSO) was gradually added drop-wise at a molar ratio of 1:10. The mixture was stirred in the dark for 15 min, followed by dilution with PBS to terminate the reaction. Unreacted fluorescent molecules were removed through ultrafiltration using a 30 kDa ultrafiltration tube and centrifugation at 14,000 g for 10 min. This process was repeated three times to recover Cy5.5-labeled Tra (Tra^Cy5.5^). Fluorescence intensity was assessed using a spectrophotometer, and the product was freeze-dried and stored at -20 °C.

### 2.12 Biodistribution of Tra

For fluorescence imaging, Tra^Cy5.5^-loaded peptide hydrogels were prepared using the aforementioned methods, except that Tra was substituted with Tra^Cy5.5^. When the tumors in nude mice reached a volume of ∼100 mm³, each mouse was peritumorally injected with 100 µl Tra^Cy5.5^-loaded hydrogels (*n* = 3 per group). Subsequently, *in vivo* imaging was performed using an IVIS Spectrum system (excitation: 670 nm, emission: 710 nm) on Day 1, 3, 5, 7, 9, 12, and 14. Mice were euthanized on Day 7 and 14 post-administration and the major organs (heart, liver, spleen, lung and kidney) were collected for observation using an IVIS imaging system. The average fluorescence intensity was quantified.

### 2.13 Measurement of serum Tra concentration

When tumor volumes reached ∼100 mm³, mice were peritumorally injected with 100 µl of different Tra-loaded hydrogel formulations. Seven days post-administration, ∼20 µl venous blood was collected from the orbital venous plexus, allowed to stand at room temperature for 2 h to clot, and centrifuged at 3000 × g for 5 min at 4 °C to separate serum. Serum Tra concentration was determined using a commercial ELISA kit following the manufacturer’s instructions.

### 2.14 Colocalization of Tra and type I collagen in tumors

When tumor volumes reached ∼100 mm³, mice were peritumorally injected with 100 µl Tra^Cy5.5^-loaded dual-affinity or Tra^Cy5.5^-loaded hydrogel. The protocol followed was as described in Section 2.9.3.

### 2.15 Cytotoxicity assessment of Tra-loaded peptide hydrogels

SKOV3 cells were seeded in the lower chamber of 12-well plates with Transwell chambers at 6 × 10⁴ cells/well, supplemented with 1.5 ml complete medium. The upper chamber received a mixture of 460 µL complete medium and 40 µl Tra-loaded hydrogels from various groups (containing 80 µg Tra). The cells were incubated for 4 d. Cell survival rate was determined by MTT assay. The absorbance at 490 nm of each well was recorded using a microplate reader (Multiskan FC, Thermo). Cell viability was calculated using the following equation:

$$\text{Viablility}\left(\%\right)=\frac{A_{\text{sample}}-A_{\text{blank}}}{A_{\text{control}}-A_{\text{blank}}}\times \;100\%$$

where *A* represents the absorbance of cells treated with assemblies or untreated cells. The half-maximal inhibitory concentration (IC_50_) values were calculated using GraphPad Prism 9, with concentration and cell viability ratio as parameters.

### 2.16 In vivo antitumor efficacy

SKOV3 tumor-bearing nude mice were employed to assess *in vivo* antitumor efficacy. When tumor volumes reached 50–60 mm³, mice were randomly divided into 5 groups (*n* = 3 per group): saline, Tra@Gel, Tra@CBP-Gel, Tra@MEP-Gel, and Tra@Dual-affinity Gel. Each group received a single peritumoral subcutaneous injection of 100 µL hydrogel containing Tra at a dosage of 10 mg/kg. Tumor growth and body weight of mice were recorded every 2 d. Tumor volumes were calculated using formula: V = Length × Width × Width / 2.

Mice were euthanized when body weight decreased by > 40% or tumor volume exceeded 1500 mm³, marking the end of the experiment. Survival duration curves were subsequently plotted. After the 28-d period, the mice were euthanized, and tumors were collected to compare tumor volumes and calculate the tumor growth inhibition rate (TGI) using the following formula: TGI (%)= (1 – V_treatment_ / V_control_) × 100%. Additionally, the tumors and major organs were obtained for histological analysis by H&E staining.

### 2.17 Blood biochemistry for assessment of liver and kidney functions

Whole blood (∼200 µl) was collected from mice before euthanasia via orbital venous plexus puncture, allowed to stand for 2 h, and centrifuged at 3000 × g for 5 min to obtain serum. The liver (alanine aminotransferase [ALT], aspartate aminotransferase [AST], albumin [ALB], total protein [TP], and alkaline phosphate [ALP]) and kidney (uric acid [UA], creatinine [CREA], and urea [UREA]) function indices were analyzed via blood biochemistry.

### 2.18 In vivo biocompatibility of hydrogels

After euthanasia, injection sites were incised to observe hydrogel retention, subcutaneous nodules, or inflammation. Tissue samples from injection sites were fixed, paraffin-embedded, sectioned, and stained with H&E to evaluate biocompatibility.

### 2.19 Statistical analysis

All quantitative experiments were independently repeated three or more times to obtain quantitative data, which are presented as mean ± standard error of the mean (mean ± SEM). Data processing and statistical analysis were conducted using Microsoft Excel or Prism 8.0 (GraphPad). Statistical significance was analyzed using one-way analysis of variance. Unless otherwise specified, *P* < 0.05 was considered statistically significant (*P < 0.05, **P < 0.01, ***P < 0.001, ****P < 0.0001).

The antitumor efficacy study used *n* = 3 per group. Post‑hoc power analysis showed power > 0.80 for the main comparisons, suggesting the observed differences are reliable despite the small sample size. Larger cohorts will be used in follow‑up studies.

## 3 Results and discussions

### 3.1 Design, self-assembly and functional affinity of peptides

**Fig. 1:**
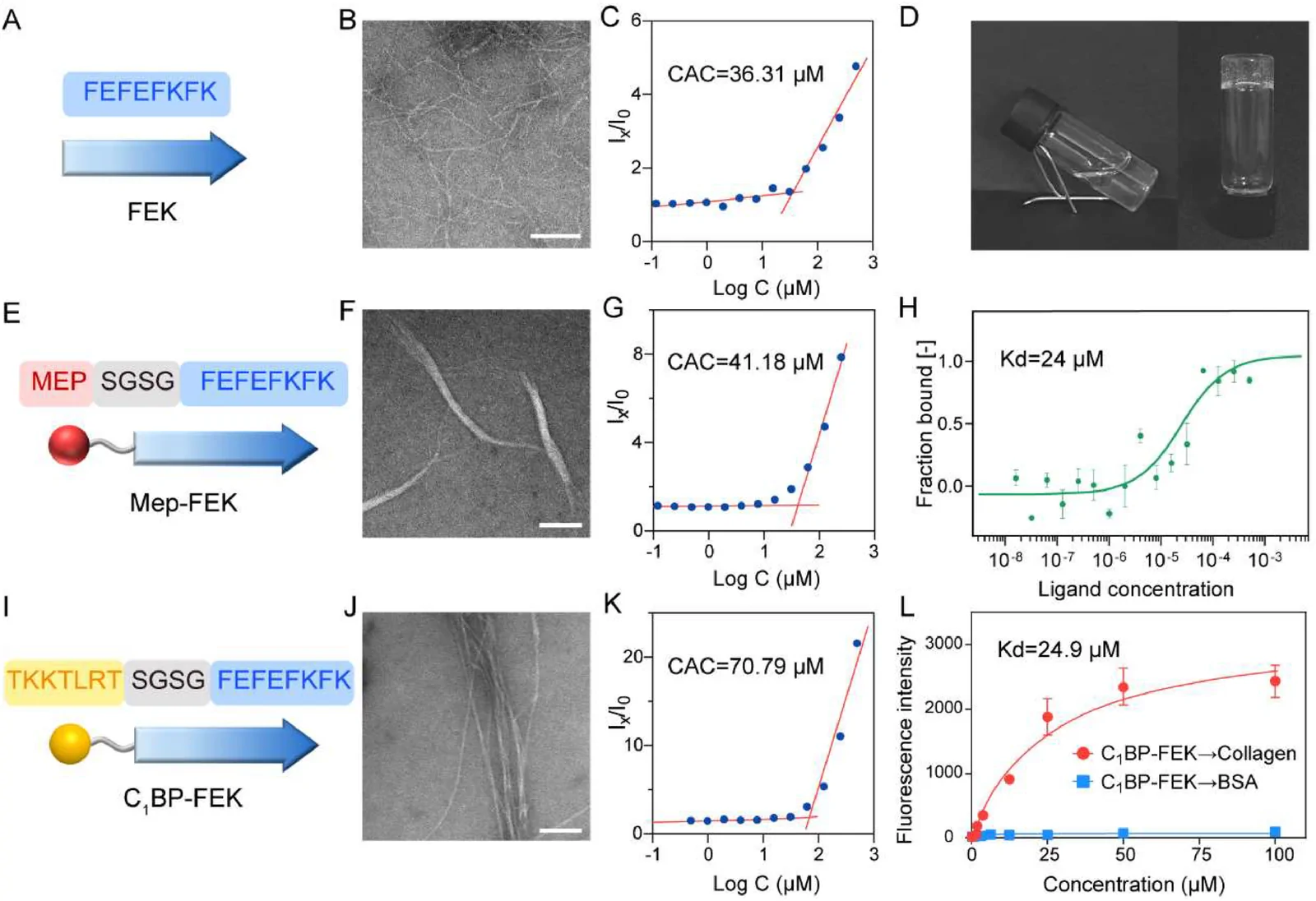
Design and characterization of hydrogel matrix, antibody affinity and collagen-binding self-assembling peptides. (A, E, I) Sequences and schematic representation of the hydrogel matrix peptide (FEK), antibody-affinity peptide (MEP-FEK) and collagen-binding peptide (C_1_BP-FEK); (B, F, J) TEM images showing the morphology of the three self-assembled peptides. Scale bar: 100 nm; (C, G, K) CAC of the three peptides; (D) Photograph showing the appearance of the FEK hydrogel; (H) MST-based assay for the binding affinity of MEP-FEK for Tra (dissociation constant, *K*_d_ = 24 µM, *n* = 3); (L) Enzyme-linked immunosorbent assay-based assessment of specific binding between C_1_BP–FEK and type I collagen (*K*_d_ = 24.9 µM; BSA was used as a negative control, *n* = 3).

Conventional antibody delivery systems are plagued by inherent limitations, including uncontrolled antibody burst release and insufficient local retention at pathological sites [16,32]. To address these bottlenecks, we engineered a dual-affinity self-assembling peptide hydrogel, integrating antibody-binding and tumor ECM-targeting motifs into a co-assembled peptide scaffold. Based on previous studies, the ionic self-complementary peptide FEFEFKFK (FEK, Fig. 1A) was selected as the hydrogel matrix, as it spontaneously assembles into β-sheet NFs under physiological conditions (pH 7.4, salt ions) [23,33]. Two functional peptides were rationally engineered based on the FEK scaffold: MEP-FEK (antibody-affinity peptide) and C_1_BP-FEK (collagen-binding peptide). MEP-FEK was engineered by conjugating 4-MEP to the FEK backbone via a flexible SGSG linker (Fig. 1E). MEP binds broadly to conserved regions of IgG via hydrophobic interactions [24]. The SGSG linker minimized steric hindrance between the affinity motif and the self-assembling backbone, preserving both β-sheet formation of FEK and MEP accessibility for antibody binding [34]. C_1_BP-FEK was constructed by incorporating the collagen-binding motif TKKTLRT into FEK via the same SGSG linker (Fig. 1I). This motif possesses cross-species specificity for type I collagen and favorable tissue permeability, thereby enabling targeted anchoring of the hydrogel to the tumor ECM [25,27,35]. All peptides were synthesized with a purity exceeding 95%, meeting the requirements for experimental use (Figs. S1–S3).

The morphological features and secondary structure of self-assembled peptides are critical determinants of their ability to form stable hydrogel scaffolds and display functional motifs, directly influencing subsequent co-assembly compatibility and biological performance [23,36]. Herein, the self-assembly behavior of three designed peptides was systematically characterized.

TEM images of peptide solutions in PBS (pH 7.4) revealed nanofibrous structures for all three peptides (Fig. 1B, 1F and 1J). The matrix peptide FEK formed uniform, well-dispersed NFs with a diameter of 6–7 nm and homogeneous length distribution, consistent with reported assembly behavior [37]. MEP-FEK NFs tended to aggregate into clusters due to MEP interactions, whereas C_1_BP-FEK exhibited lower fiber density.

The self-assembled NF-forming capacity of these peptides was verified using CAC. FEK exhibited a low CAC value of 36.31 µM (Fig. 1C), indicating a potent self-assembly capacity. MEP-FEK exhibited a comparable CAC (Fig. 1G), whereas C_1_BP-FEK exhibited a slightly higher CAC of 70.79 µM (Fig. 1K), probably due to increased hydrophilicity and protonation of Lys/Arg residues in the TKKTLRT motif at neutral pH, which weakened intermolecular electrostatic or hydrophobic interactions.

To verify the secondary structure of the assembled peptides, circular dichroism (CD) spectroscopy was employed. CD spectra of all peptides exhibited a negative peak at 216–218 nm and a positive peak at 195–197 nm (Fig. S6), indicative of β-sheet conformation, critical for peptide co-assembly [29]. The negative peak at 200 nm was attributed to the interaction of aromatic groups, consistent with previous reports [37].

Antibody- and collagen-binding affinities are core determinants of the performance of dual-affinity systems in mediating sustained antibody release and prolonged tumor retention, requiring quantification via *K*_d_ measurements. MST revealed that MEP-FEK bound Tra with a *K*_d_ of 24 µM (Fig. 1H), comparable to reported values for antibody-affinity systems [12]. The ELISA results validated the specific binding of C_1_BP-FEK to type I collagen (*K*_d_ = 24.9 µM), which was sufficient for stable anchoring of the hydrogel to the tumor ECM. Notably, C_1_BP-FEK exhibited negligible non-specific binding to BSA, confirming its robust collagen-binding specificity (Fig. 1L).

Gelation capacity, a core property, directly determines the feasibility of using peptide scaffolds for controlled drug delivery. FEK exhibited the ability to form transparent hydrogels at pH ≤ 7 and concentrations ≥ 5 mM, and PBS-induced charge shielding triggered rapid sol-gel transitions, which confirmed the suitability of FEK as a hydrogel matrix (Fig. 1D). MEP-FEK and C_1_BP-FEK maintained gelation potential yet relied on co-assembly with FEK to stabilize fibrous networks (Fig. S7).

### 3.2 Peptide co-assembly ratio optimization and nanofiber characterization

The ratio of functional peptides (MEP-FEK and C_1_BP-FEK) and matrix peptide (FEK) is critical for balancing the dual-affinity functionality and hydrogel structural stability. Excessive functional peptides lead to overcrowding of functional domains, inducing steric hindrance with target proteins and impairing effective interactions [38]. Moreover, overly dense functional group distribution may also compromise hydrogel mechanical properties via electrostatic interactions [39]. Conversely, insufficient amounts fail to achieve the desired binding efficiency.

**Fig. 2:**
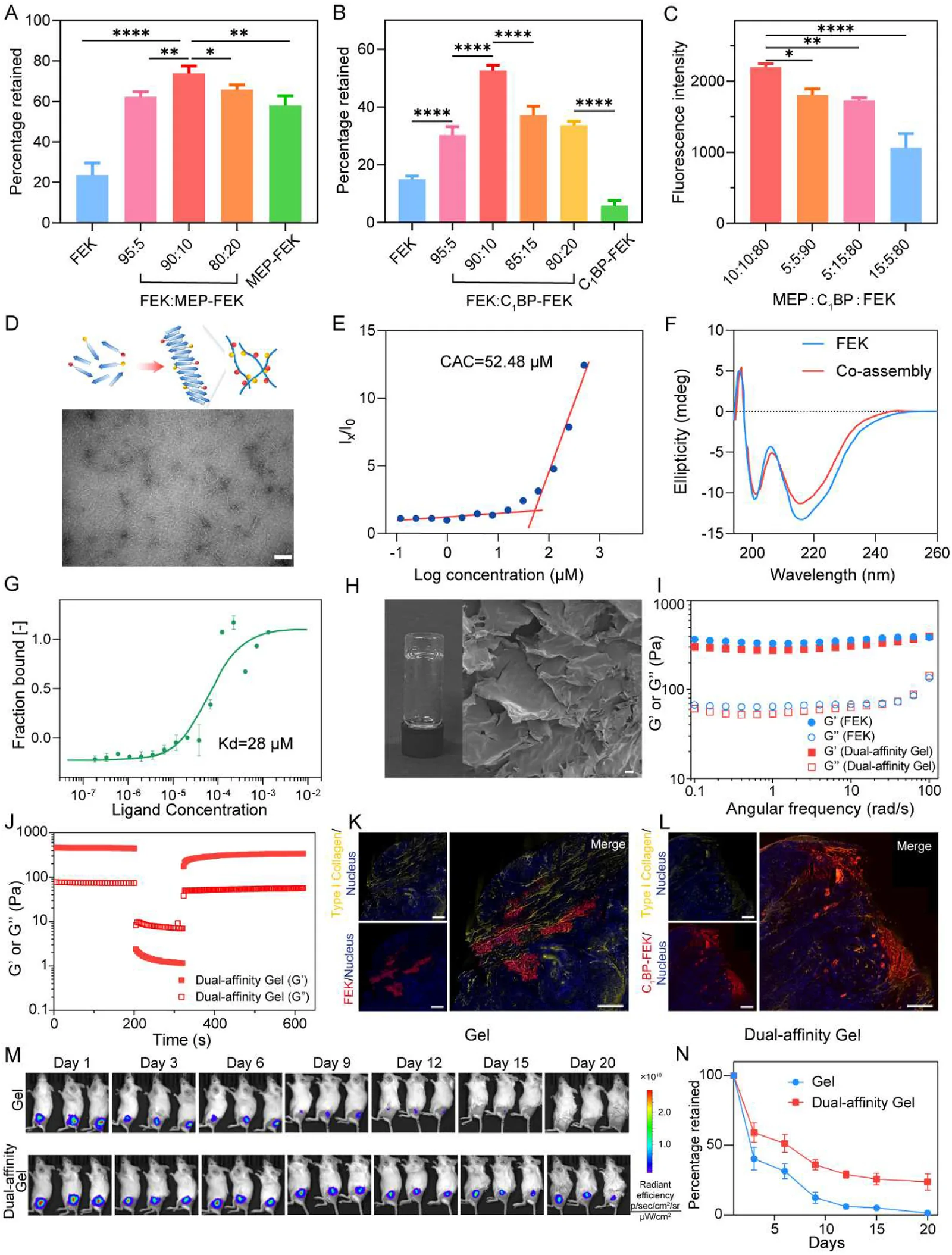
Optimization of the co-assembly ratios and *in vitro/in vivo* characterization of dual-affinity peptide co-assembly systems. (A) IgG retention rate of FEK/MEP-FEK co-assemblies at varying molar ratios (*n* = 3, *P < 0.05, **P < 0.01, ****P < 0.0001); (B) Type I collagen retention rate of FEK/C_1_BP-FEK co-assemblies at varying molar ratios (*n* = 3, ****P < 0.0001); (C) IgG retention capacity of ternary FEK/MEP-FEK/C_1_BP-FEK co-assemblies at various ratios, determined by ELISA-based assay (*n* = 3, *P < 0.05, **P < 0.01, ****P < 0.0001); (D) Schematic illustration and TEM-characterized morphology of dual-affinity ternary peptide co-assembled nanofibers; (E) CAC of co-assembled nanofibers determined using ThT fluorescence assay; (F) CD spectra of FEK self-assemblies and dual-affinity peptide co-assemblies; (G) Binding kinetic curve of dual-affinity co-assemblies with Tra, yielding a *K*_d_ of 28 µM (*n* = 3); (H) Appearance of dual-affinity peptide hydrogel and scanning electron microscopy image of lyophilized dual-affinity peptide hydrogel; (I, J) Frequency sweep and dynamic step-strain oscillation test results of non-affinity hydrogel and co-assembled dual-affinity peptide hydrogel; (K, L) Immunofluorescence staining of type I collagen (yellow) co-localized with FEK/C_1_BP-FEK peptides (red) in non-affinity Gel and Dual-affinity Gel, nuclei are stained blue. Scale bar: 700 µm; (M) *In vivo* fluorescence imaging of local retention for non-affinity Gel and Dual-affinity Gel in mice over 20 d (*n* = 3); (N) Quantitative analysis of fluorescence retention over time (*n* = 3).

First, affinity capture assays were used to determine the optimal molar ratios of MEP-FEK:FEK and C_1_BP-FEK:FEK individually. For MEP-FEK:FEK, the co-assembled system with a ratio of 1:9 achieved the highest IgG retention (> 74%), markedly higher than that of the non-affinity FEK hydrogel (∼20%) and standalone MEP-FEK (∼60%) (Fig. 2A). This indicated that the matrix peptide FEK enhanced the antibody-binding efficiency of MEP-FEK by promoting uniform fiber formation. The resultant hydrogel was designated MEP-Gel. For C_1_BP-FEK:FEK, FITC-labeled type I collagen was used as the target. At a ratio of 1:9, over 52% collagen retention was achieved, whereas standalone C_1_BP-FEK showed < 10% retention due to poor self-assembly capacity at physiological pH (Fig. 2B), confirming the critical role of FEK in improving the assembly and collagen-binding performance of C_1_BP-FEK. This co-assembled hydrogel was designated C_1_BP-Gel.

Subsequently, an ELISA-based method was established to screen the ternary co-assembly ratio (MEP-FEK:C_1_BP-FEK:FEK). Type I collagen was pre-coated on 96-well plates to mimic the tumor ECM microenvironment, then incubated with FITC-labeled IgG-containing hydrogels to simulate the hydrogels’ *in vivo* behavior in tissue environments. Post-wash fluorescence intensity reflected the antibody retention capacity, a direct indicator of dual-affinity synergy. As shown in Fig. 2C, the hydrogel with a molar ratio of 1:1:8 exhibited the highest IgG retention. This ratio was thus determined as the optimal formulation for dual-affinity hydrogels (Dual-affinity Gel), as it balanced the exposure of antibody-affinity and collagen-binding motifs while maintaining stable co-assembly.

TEM, CAC and CD measurements were utilized for comprehensive characterization of peptide co-assemblies. TEM imaging (Fig. 2D) revealed that the ternary co-assembly formed uniform, well-dispersed long NFs with a diameter of 6–7 nm, consistent with that of the matrix peptide FEK (Fig. 1B). In contrast, physical mixing of individual peptide solutions yielded heterogeneous fibers with irregular lengths and diameters (Fig. S8). The uniform nanofibrous morphology stemmed from the conserved FEK motif across all three peptides, which promoted intermolecular hydrogen bonding and β-sheet formation. As shown in Fig. 2E, the CAC of the co-assembled system was 52.48 µM, which was slightly higher than that of FEK and MEP-FEK alone but lower than that of C_1_BP-FEK. This suggests that incorporating self-assembling FEK mitigated the adverse effects on assembly performance resulting from the functional domain coupling, and the co-assembly strategy facilitated a stable affinity peptide-FEK matrix system. CD spectroscopy of co-assembled NFs exhibited a negative peak at 217–218 nm and a positive peak at 195–197 nm, characteristic of a β-sheet secondary structure (Fig. 2F). This spectral profile was consistent with that of individual FEK NFs, indicating that incorporation of MEP-FEK and C_1_BP-FEK did not disrupt the β-sheet conformation, which is an essential prerequisite for peptide self-assembly and hydrogel formation [40]. Additionally, co-assembly with FEK eliminated spectral peak discrepancies of the two affinity peptide NFs, suggesting that FEK effectively eliminates the self-assembly of individual affinity peptides, thereby facilitating their ordered organization and efficient co-assembly.

To characterize the binding affinity of the co-assembled system for Tra, the *K*_d_ was determined via MST. The measured *K*_d_ value was 28 µM (Fig. 2G), which was comparable to that of standalone MEP-FEK (24 µM) and consistent with reported values for antibody-affinity delivery systems [12]. This indicated that co-assembly with FEK did not significantly compromise the antibody-binding capacity of MEP-FEK, as the flexible SGSG linker minimized steric hindrance and ensured the exposure of MEP motifs on the fiber surface.

### 3.3 Morphological, rheological characterization and injectability of co-assembled hydrogels

Scanning electron microscopy (SEM) images of lyophilized hydrogels revealed a lamellar ordered stacking structure for both the dual-affinity and non-affinity FEK hydrogels (Figs. 2H and S9). No significant morphological differences were observed between the two, suggesting that the incorporation of the affinity peptide into the co-assembly system did not alter the overall structure of the hydrogel.

Rheological characterization was performed to evaluate the structural stability and injectability of the dual-affinity hydrogel. Frequency sweep tests showed that the storage modulus (*G*′) of the dual-affinity hydrogel (309.46 Pa) was slightly lower than that of the non-affinity FEK hydrogel (357.13 Pa) but remained significantly higher than the loss modulus (*G*″) across the frequency range of 0.1–100 rad/s (Fig. 2I), indicating a stable gel structure and favorable mechanical properties for drug delivery. The 3ITT further confirmed its shear-thinning and self-healing properties. When the strain was increased from 1% to 100% to simulate injection shear stress, *G*′ rapidly decreased below *G*″ (liquid-like behavior). Upon strain recovery to 1%, *G*′ and *G*″ recovered to their initial values within seconds (Fig. 2J). This rheological profile confirmed the hydrogel’s injectability, as it can flow through a syringe under shear force and rapidly recover its gel-like structure at the injection site. The above data were obtained from blank hydrogels. To verify whether the 2 mg/mL Tra loading disrupts the peptide network, we measured the rheological properties of the drug‑loaded dual‑affinity hydrogel. The frequency sweep showed *G*′ >> *G*″ across 0.1–100 rad/s, and the 3ITT test demonstrated rapid shear‑thinning and full recovery (Fig. S10), confirming that the high antibody payload does not compromise gel stability or injectability.

To further directly demonstrate injectability and dosage uniformity, each hydrogel formulation was loaded into a 1 mL syringe and manually expelled. As shown in Fig. S11, the dual‑affinity hydrogel smoothly exited the needle and formed a continuous, self-supporting gel immediately upon contacting the culture dish surface. Quantitative analysis of the expelled mass (Table S1) revealed a CV below 3% for all formulations, indicating excellent dosage uniformity across repeated injections. These results confirm that the dual‑affinity hydrogel can be reliably administered via standard syringe injection, consistent with the rheological shear‑thinning and self‑healing properties described above.

### 3.4 Collagen-binding capacity and in vivo retention

Immunohistochemistry confirmed the abundant expression of type I collagen in subcutaneous SKOV3 tumor xenografts, with brown-yellow staining indicating its expression, which was predominantly localized within and around tumors (Fig. S12). This provided a biological basis for the collagen-targeting design of the hydrogel. To verify that the prolonged retention was mediated by specific binding to type I collagen, immunofluorescence co-localization assays were performed. Cy5.5-labeled C_1_BP-FEK was incorporated into the dual-affinity hydrogel, followed by peritumoral injection into SKOV3-bearing nude mice. Frozen sections of tumor tissues exhibited strong co-localization between Cy5.5-labeled C_1_BP-FEK (red) and Cy3-labeled type I collagen (yellow) (Fig. 2L). In contrast, the Cy5.5-labeled FEK exhibited almost no co-localization with Cy3-labeled type I collagen (Fig. 2K). These results confirmed that the C_1_BP-FEK motif enabled specific anchoring of the hydrogel to the tumor ECM via targeted binding to type I collagen.

*In vivo* retention of the dual-affinity hydrogel was assessed by subcutaneous injection of Cy5.5-labeled FEK-based dual-affinity hydrogel into BALB/c mice. As shown in Fig. 2M and 2N, dual-affinity hydrogels exhibited significantly higher fluorescence intensity than non-affinity hydrogels, maintaining levels approximately 10%–20% higher levels throughout the 19-d post-injection observation period. By 19 d, non-affinity hydrogels showed almost complete fluorescence loss, indicative of near-total subcutaneous clearance, whereas collagen-affinity hydrogels retained ∼12% of their initial fluorescence intensity. These results confirmed that the incorporation of collagen-affinity peptides substantially enhances hydrogel subcutaneous retention. The dual‑affinity hydrogel is slowly biodegradable, and its prolonged retention relies on both slow degradation and C_1_BP‑mediated anchoring to collagen I. Local injection alone is insufficient, as non‑affinity gels are rapidly cleared.

### 3.5 In vitro release of antibody-loaded dual-affinity hydrogels

**Fig. 3:**
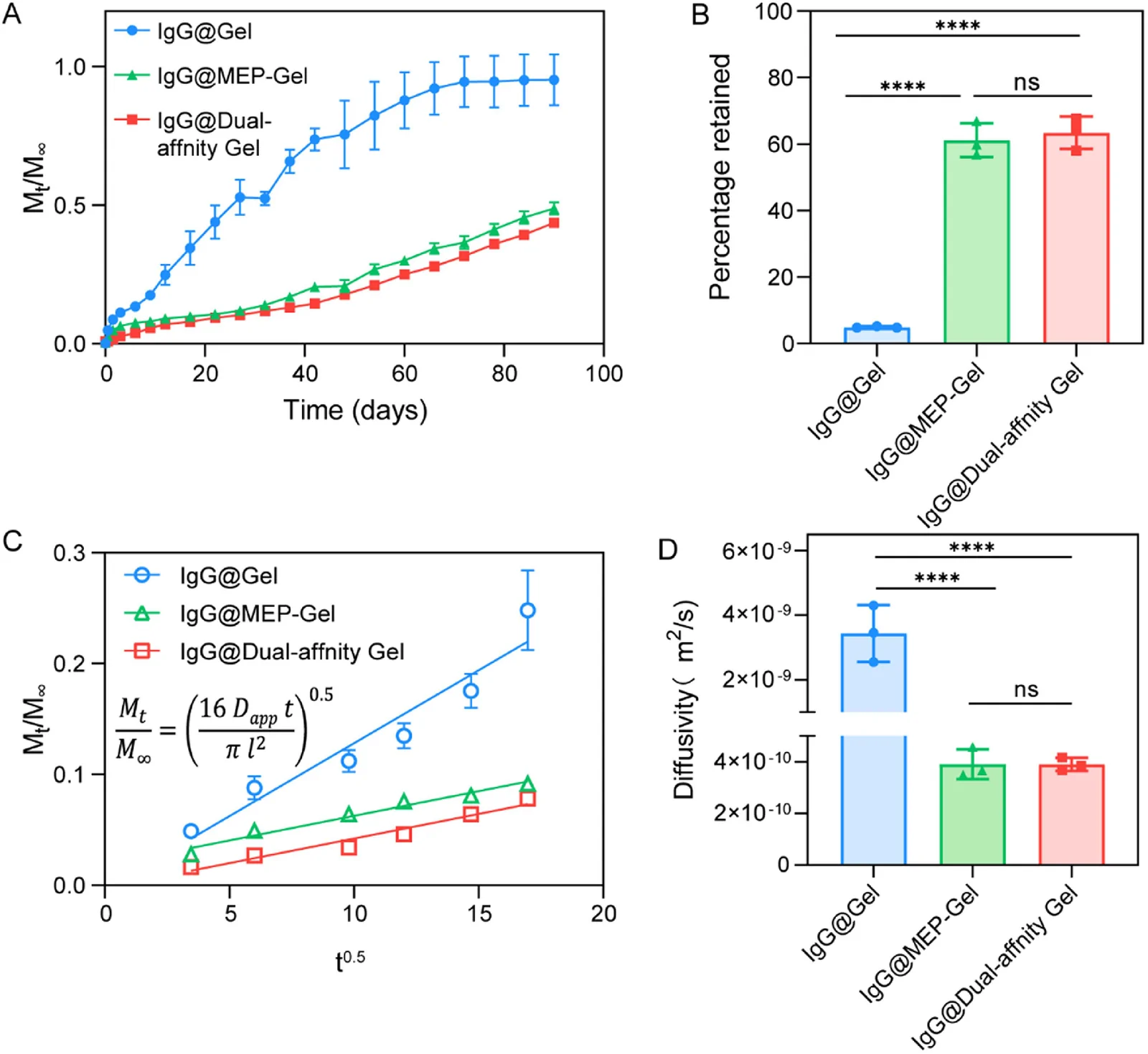
*In vitro* release behavior of IgG-loaded hydrogels. (A) Cumulative IgG release profiles of IgG@Gel, IgG@MEP-Gel and IgG@Dual-affinity Gel over 90 d, expressed as M_t_/M_∞_ (fraction of total released IgG) (*n* = 3); (B) Percentage of IgG retained in hydrogels after 90 days of *in vitro* release (*n* = 3, ****P < 0.0001, ns: not significant); (C) Evaluation of apparent diffusion coefficient (D_app_) based on the first 6 time points (*n* = 3); (D) Quantitative comparison of D_app_ among hydrogel groups, reflecting initial IgG release rate (*n* = 3, ****P < 0.0001, ns: not significant).

The dual-affinity hydrogel theoretically exhibits almost universal compatibility with therapeutic antibodies. For preliminary proof-of-concept investigations, the widely used IgG was selected as a model antibody for the release study. Fig. S13 shows the macroscopic appearance of antibody-loaded hydrogel, which self-assembled into a highly viscous, non-flowable solid in vial and retained structural integrity without flow upon inversion. As shown in Fig. 3A and 3B, significant differences in antibody release profiles were observed between affinity and non-affinity hydrogels. Non-affinity hydrogels ceased antibody release by 70 d, with ∼95% cumulative release on Day 90. In contrast, affinity hydrogels exhibited slower sustained release, with merely ∼45% cumulative release over the same period. These results confirm the superior capacity of affinity hydrogels for sustained and controlled antibody release, which is attributed to the incorporation of the MEP-FEK peptide into the hydrogel structure. Dual-affinity hydrogels displayed a comparable release profile to antibody-affinity hydrogels, confirming that C_1_BP incorporation did not impair the antibody-affinity ligand function.

The apparent diffusion coefficient (D_app_) further validated the affinity-mediated controlled release mechanism. As shown in Fig. 3C, linear fitting of the M_t_/M_∞_-t^0.5^ plot based on the first six time points yielded good linearity (R² = 0.88–0.95) for all groups. Quantitative analysis revealed that the slope (k = 0.01316) of the non-affinity hydrogel group was 2.97-times that of the two affinity hydrogel groups (MEP-Gel and Dual-affinity Gel, k = 0.00445), indicating that the D_app_ of antibody in non-affinity hydrogels was 8.8 times higher than that in affinity hydrogels (Fig. 3C and 3D). Further calculation of the diffusion rate revealed that the IgG diffusivity from non-affinity hydrogels was 3.43 ± 0.9 × 10^–9^ m^2^/s, which was significantly reduced to 3.9 ± 0.6 × 10^–10^ m^2^/s in hydrogels functionalized with antibody-affinity ligands, corresponding to a nearly one-order-of-magnitude reduction. This significant decrease in diffusivity confirms the efficacy of affinity ligands in controlling antibody release. Beyond enabling sustained long-term release, the affinity hydrogels also exhibited a pronounced attenuation of antibody release in the initial phase, endowing the system with considerable potential to circumvent the toxicological risks associated with burst release in conventional hydrogels. The release of antibody is governed by both dissociation of the MEP‑antibody interaction and gradual network degradation; MEP density and ECM anchoring are key parameters that can be tuned to modulate the release kinetics.

### 3.6 In vivo retention and biodistribution of antibody-loaded dual-affinity hydrogels

**Fig. 4:**
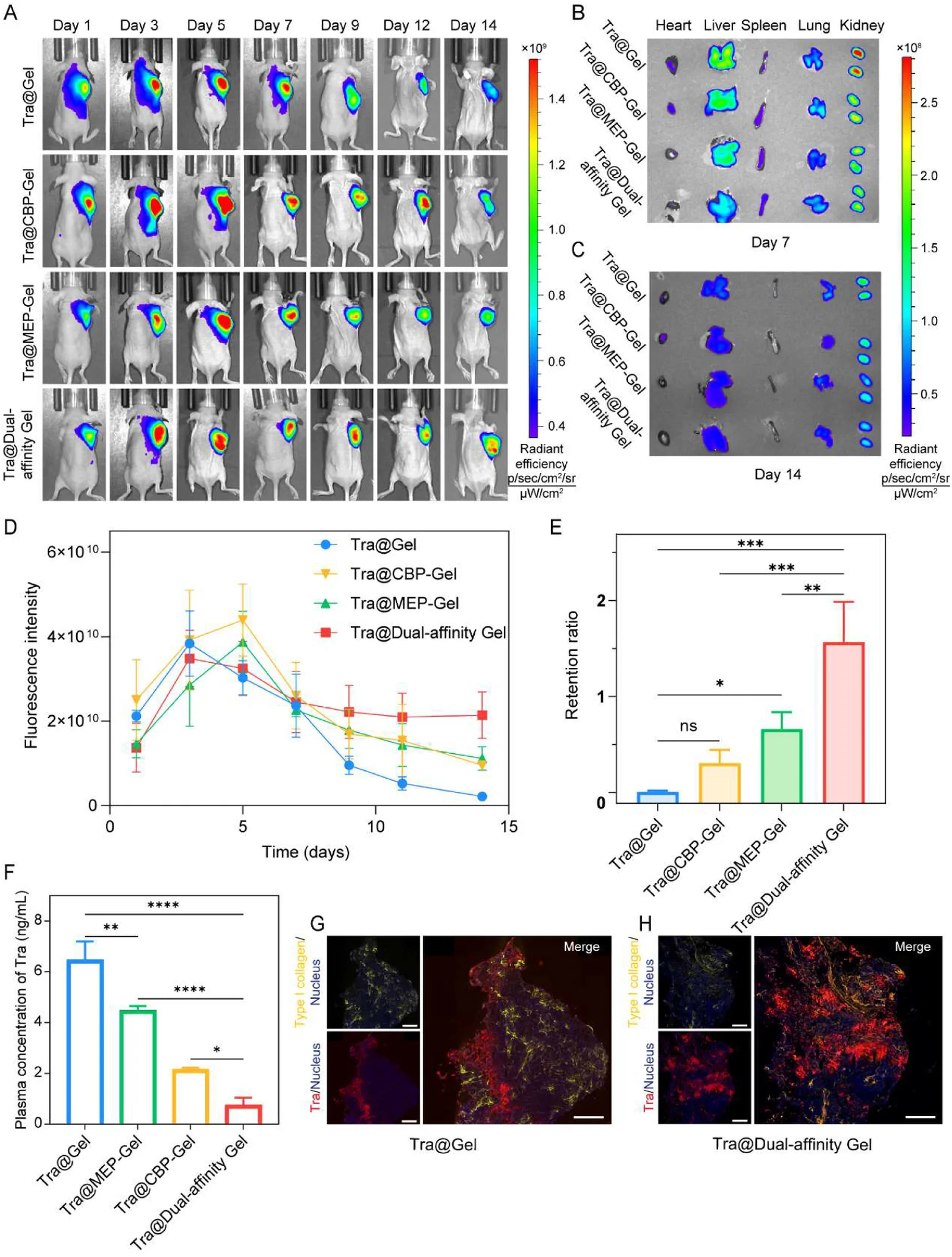
*In vivo* tumor retention and biodistribution of Tra-loaded hydrogels. IVIS *in vivo* fluorescence images (A) and quantitative analysis (D) of local tumor retention of Tra in various hydrogel groups (Tra@Gel, Tra@CBP-Gel, Tra@MEP-Gel and Tra@Dual-affinity Gel) on Day 1–14 post-administration (*n* = 3); *Ex vivo* fluorescence imaging of Tra distribution in major organs (heart, liver, spleen, lungs, and kidneys) from various hydrogel groups on Day 7 (B) and 14 (C) post-administration; (E) Percentage of Tra fluorescence retention percentage in tumors relative to the initial amount in different hydrogel groups on Day 14 post-administration (*n* = 3, *P < 0.05, **P < 0.01, ***P < 0.001); (F) Serum Tra concentration in mice from different hydrogel groups on Day 7 post-administration (*n* = 3, *P < 0.05, **P < 0.01, ****P < 0.0001). Immunofluorescence co-localization of Tra (red) and type I collagen (yellow) in tumor tissues from the Tra@Gel (G) and Tra@Dual-affinity Gel (H) groups. Scale bar: 700 µm.

To evaluate the *in vivo* retention of the hydrogel system, Cy5.5-labeled Tra (Tra^Cy5.5^) was loaded into hydrogels, and peritumorally injected into SKOV3-bearing nude mice. An IVIS small animal imaging system was employed to track the local retention and *in vivo* distribution of the antibody. As shown in Fig. 4A and 4D, the fluorescence signal of non-affinity Tra@Gel declined most rapidly and was barely detectable by 14 d, indicating rapid clearance of the antibody. In contrast, both antibody-affinity and collagen-affinity hydrogels exhibited a markedly gradual decrease in fluorescence intensity, indicating a prolonged release profile. Quantitative analysis (Fig. 4E) revealed that tumor-site antibody fluorescence retention in the non-affinity group was ∼12% of the initial dose on Day 14, whereas the retention rates reached 42% and 78% for the collagen-affinity and antibody-affinity groups, respectively. Notably, the dual-affinity hydrogel exhibited the most sustained release profile and the highest fluorescence intensity at the tumor site on Day 14, with its tumor antibody fluorescence retention even reaching 1.8-fold of that for the initial dose. These results confirmed that, in contrast to non-affinity hydrogels, the two single-affinity hydrogels achieved sustained local antibody release via antibody affinity and tumor matrix adhesion, respectively. Evidently, the dual-affinity hydrogel integrates these two functional attributes to confer the most superior sustained release performance and markedly enhanced tumor antibody retention. Additionally, the lower fluorescence signal observed for the dual‑affinity hydrogel on Day 1 is attributed to concentration‑dependent self‑quenching of Cy5.5 within the gel depot, which is alleviated upon gel hydration (Fig. S14).

Fluorescence imaging on Day 7 and 14 showed higher antibody accumulation in major organs in the non-affinity hydrogel group, indicating greater systemic exposure (Fig. 4B and 4C). In contrast, the dual-affinity hydrogel group exhibited significantly lower fluorescence in major organs, suggesting reduced off-target distribution. Consistent with the tissue distribution results, at 1-week post-administration, the non-affinity hydrogel group exhibited the highest serum levels of circulating Tra (Fig. 4F), indicative of extensive drug release and systemic diffusion. In contrast, the dual-affinity hydrogel group showed significantly lower serum Tra concentrations, suggesting enhanced local antibody retention and reduced systemic leakage. These results verify that the dual-affinity hydrogel system effectively enhanced local antibody retention while minimizing systemic exposure, potentially enhancing therapeutic efficacy and minimizing off-target effects.

To validate the mechanistic basis for prolonged tumor-localized antibody retention, immunofluorescence co-localization assays were conducted to characterize the spatial association between Tra and type I collagen in tumor tissues. Compared with the non-affinity hydrogel group, the dual-affinity hydrogel group exhibited significant co-localization of red fluorescence-labeled Tra and yellow fluorescence-labeled collagen type I within the tumor (Fig. 4G and 4H). These results suggest that the C_1_BP-FEK and MEP-FEK in the dual-affinity hydrogel mediate specific affinity interactions: C_1_BP-FEK effectively anchors the hydrogel scaffold to type I collagen in the tumor ECM, while MEP-FEK retains the loaded antibody within the hydrogel network. Collectively, these interactions prolong the retention of Tra at the tumor site by preventing rapid hydrogel clearance and antibody diffusion.

### 3.7 Antitumor efficacy of Tra-loaded dual-affinity hydrogels

**Fig. 5:**
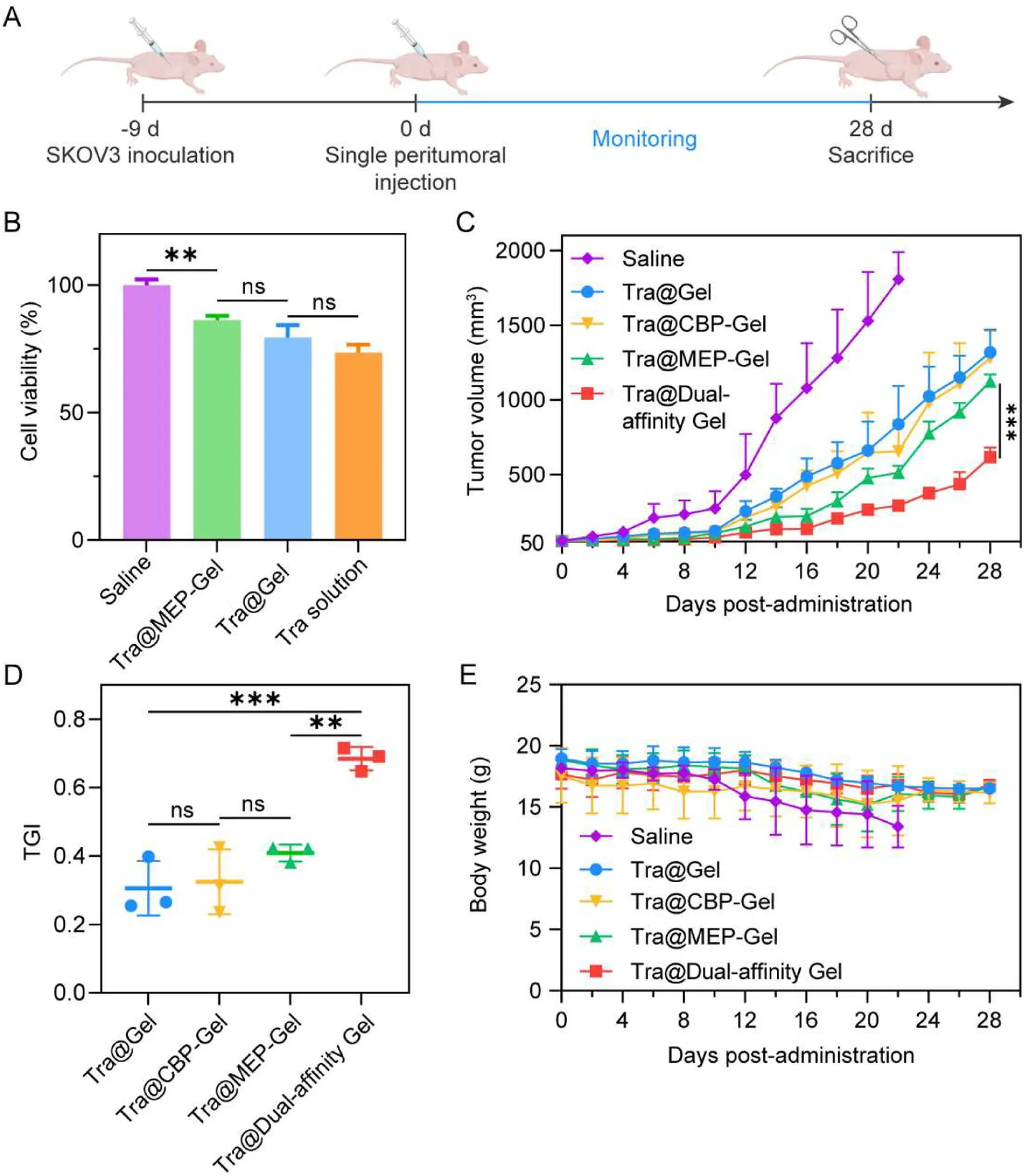
*In vitro* cytotoxicity and *in vivo* antitumor efficacy of Tra-loaded hydrogels. (A) Schematic illustration of *in vivo* antitumor experiment schedule. Created with BioGDP.com; (B) *In vitro* cytotoxicity of different formulations against SKOV3 cells (*n* = 3, **P < 0.01); (C) Tumor growth curves following single peritumoral injection of different formulations (*n* = 3, ***P < 0.001); (D) TGI rates of different formulation groups on Day 28 post-administration (*n* = 3, **P < 0.01, ***P < 0.001); (E) Body weight change curves for mice over the treatment period (*n* = 3). Note: The saline control group was terminated prematurely on Day 22 due to tumor volume/body weight approaching animal ethical endpoints, whereas all other groups were maintained until the scheduled endpoint on Day 28. The TGI values are based on the saline control values estimated by last observation carried forward (LOCF).

To confirm whether the dual-affinity design can translate to superior antibody-based antitumor efficacy, we evaluated the *in vitro* and *in vivo* antitumor efficacy. It should be noted that the SKOV3 xenograft model is used here primarily as a platform to assess hydrogel performance *in vivo*, rather than to assess the clinical therapeutic outcome of Tra against ovarian cancer. For *in vitro* assessment, cytotoxicity assays were performed on human ovarian cancer SKOV3 cells. Free Tra exhibited weak inherent cytotoxicity (Fig. 5B), as its primary mode of action is antibody-dependent cellular cytotoxicity (ADCC). Notably, the antibody-affinity hydrogel showed weaker cytotoxicity toward SKOV3 cells than the non-affinity hydrogel and free Tra groups. Consistent with *in vitro* release data, the sustained release property of the antibody-affinity hydrogel resulted in lower Tra concentrations in the culture medium than the non-affinity counterpart at the same time points, reducing the dose-dependent pharmacodynamic effect. Nevertheless, measurable cytotoxicity was retained, confirming that the hydrogel matrix does not compromise the inherent bioactivity of Tra.

The *in vivo* antitumor efficacy was assessed in a SKOV3 cell xenograft mouse model. Mice received a single peritumoral injection of each formulation (Fig. 5A). The saline group was terminated on Day 22 due to body weight and excessive tumor volume approaching animal ethical endpoints. As shown in Figs. 5C, 5D and S15, all hydrogel formulations demonstrated superior therapeutic efficacy. The Tra-loaded dual-affinity hydrogel exhibited the most pronounced antitumor activity, achieving an inhibition rate of 68%. Tumor growth curves revealed distinct therapeutic profiles across hydrogel groups. At 2 weeks post-administration, both the antibody-affinity and dual-affinity hydrogel groups exhibited significantly enhanced antitumor effects compared with those of the non-affinity hydrogel group. By comparison, the collagen-affinity hydrogel showed comparable efficacy to the non-affinity hydrogel group. Combined with the results of small-animal *in vivo* imaging, this differential activity at the 2-week time point was attributed to the antibody-affinity peptide-mediated sustained release of Tra, which prolonged the local antitumor effect of the antibody. Notably, at 28 d post-administration, only the dual-affinity hydrogel group maintained a statistically significant difference in antitumor efficacy relative to all other groups. These results further underscored the critical synergistic role of the combination of the collagen-affinity peptide and the antibody-affinity peptide, which enhanced local drug retention at the tumor site and thereby sustained and amplified the therapeutic efficacy of Tra over a prolonged timeframe.

### 3.8 Preliminary in vivo safety assessment

The body weight of nude mice was monitored over the treatment period to assess the systemic toxicity of each formulation. The saline control group exhibited a significant reduction in weight, whereas no significant differences in body weight change were noted among the hydrogel formulation groups (Fig. 5E). These results indicated that the Tra-loaded hydrogels had no inherent toxicity and alleviated tumor-associated severe weight loss in tumor-bearing mice, further supporting their favorable preliminary safety profile.

To further assess the *in vivo* biosafety, histopathological analysis (H&E staining) of major organs and subcutaneous tissues was conducted. For major organs (Fig. S16), inflammatory infiltrates were observed in the liver and lungs in the saline and non-antibody-affinity hydrogel groups, accompanied by liver nodules of uncertain origin (possibly extramedullary hematopoiesis or metastatic lesions). In contrast, no obvious toxicity or tissue lesions were detected in the antibody-affinity and dual-affinity hydrogel treatment groups. Additionally, H&E staining of subcutaneous tissues (Fig. S17) showed normal tissue morphology compared with that in the PBS control group, with no signs of accumulation or nodule formation and only minimal cutaneous inflammation. Collectively, these results indicate that the Tra‑loaded hydrogels are well tolerated in nude mice under the tested conditions, with no obvious toxicity or local inflammation.

To evaluate the hepatic and renal safety, serum biochemical assays were conducted on day 28 post-treatment. The hepatic function parameters are summarized in Fig. S15. Most hepatic and renal function indicators, including ALB, TP, UA and CREA, remained within normal reference ranges, demonstrating the favorable safety profile of the hydrogel formulation. Sporadic elevation in ALT and AST in individual model mice was plausibly attributed to liver toxicity induced by tumor metastasis, rather than to the hydrogel itself. Notably, UA and UREA levels in the antibody-affinity and dual-affinity hydrogel groups were closer to baseline values than those in other hydrogel groups, suggesting that the sustained antibody release profile of these formulations may further alleviate potential renal functional disturbances. Collectively, these biochemical evaluations confirmed that affinity-based controlled-release hydrogels exerted no significant hepatotoxicity or nephrotoxicity, and could even reduce off-target adverse effects on visceral organs, thereby improving the overall safety of Tra-mediated therapy.

## 4 Conclusions

In this study, we developed an injectable dual-affinity self-assembling peptide hydrogel for localized antibody delivery to address the key limitations of the current mAb delivery strategies, such as systemic toxicity, poor local retention, and uncontrolled burst release. By co-assembling the matrix peptide FEK with two functional peptides (MEP-FEK for non-covalent antibody binding and C_1_BP-FEK for tumor collagen targeting) at an optimized molar ratio of 1:1:8, the hydrogel formed stable β-sheet nanofibrous networks with shear-thinning and self-healing properties, enabling facile peritumoral injection and rapid *in situ* gelation. *In vitro* studies confirmed its superior sustained release performance while preserving mAb bioactivity, and *in vivo* evaluations using SKOV3 xenograft models validate enhanced local antibody retention, minimized systemic exposure, and superior antitumor efficacy with negligible organ toxicity. This dual-affinity peptide hydrogel overcomes the drawbacks of conventional hydrogels, exhibits broad application potential for various IgG antibodies, and provides a versatile, promising and translatable strategy to improve the efficacy and safety of localized antibody-based cancer therapy.

## CRediT authorship contribution statement

**Weiping Cui:** Writing - review & editing, Writing - original draft, Visualization, Validation, Project administration, Methodology, Investigation, Formal analysis, Data curation. **Limin Zhang:** Validation, Project administration, Methodology, Investigation, Formal analysis, Data curation. **Chang Yang:** Investigation. **Bing He:** Formal analysis. **Hua Zhang:** Formal analysis. **Xueqing Wang:** Formal analysis. **Zhiwen Zhang:** Formal analysis. **Qiang Zhang:** Supervision, Funding acquisition, Formal analysis, Data curation, Conceptualization. **Wenbing Dai:** Writing - review & editing, Visualization, Supervision, Resources, Project administration, Methodology, Funding acquisition, Formal analysis, Data curation, Conceptualization.

## Conflicts of interest

The authors declare that there are no conflicts of interests.

## Appendix Supplementary materials

Image, application 1
