# Supplementary material for: Injectable dual-affinity self-assembling peptide hydrogels for local controlled delivery of therapeutic antibodies
Source: Asian J Pharm Sci. 2026 Aug 12;21(4):101189. doi: 10.1016/j.ajps.2026.101189 (PMC13543808; doi:10.1016/j.ajps.2026.101189)
Supplement: Supplementary file 1 [file mmc1.docx]

**Supplementary Material**

**Injectable dual-affinity self-assembling peptide hydrogels for local controlled delivery of therapeutic antibodies**

Weiping Cui^a,#^, Limin Zhang^a,#^, Chang Yang^a^, Bing He^a^, Hua Zhang^a^, Xueqing Wang^a^, Zhiwen Zhang^b^, Qiang Zhang^a,^*, and Wenbing Dai^a,^*

**Fig. S1.** Molecular structure, ESI (+) Mass spectra and HPLC spectrum of FEK.

**Fig. S2.** Molecular structure, ESI (+) Mass spectra and HPLC spectrum of MEP-FEK.

**Fig. S3.** Molecular structure, ESI (+) Mass spectra and HPLC spectrum of C_1_BP-FEK.

**Fig. S4.** ESI (+) Mass spectra and HPLC spectrum of FEK^Cy5.5^.

**Fig. S5.** ESI (+) Mass spectra and HPLC spectrum of C_1_BP-FEK^Cy5.5^.

**Fig. S6.** Circular dichroism (CD) spectra of FEK (A), MEP-FEK (B) and C_1_BP-FEK (C) self-assembled nanofibers.

**Fig. S7.** Appearance of hydrogels formed by co-assembly of (A) MEP-FEK/FEK and (B) C_1_BP-FEK/FEK.

**Fig. S8.** TEM image of nanofibers formed by separate preparation of FEK, MEP-FEK, C_1_BP-FEK solutions and subsequent direct mixing. Scale bar: 100 nm.

**Fig. S9.** SEM image of non-affinity peptide hydrogel after lyophilization. Scale bar: 10 μm.

**Fig. S10.** Frequency sweep (A) for trastuzumab‑loaded (2 mg/mL) non-affinity hydrogel and dual-affinity hydrogel and dynamic step-strain oscillation test (B) results for trastuzumab‑loaded (2 mg/mL) dual-affinity hydrogel.

**Fig. S11.** Injectability assessment of the dual‑affinity hydrogel. Representative photograph showing extrusion of the hydrogel formulation from a 1 mL syringe. The extruded material immediately formed a continuous, self-supporting gel upon contacting the culture dish surface, confirming favorable injectability and *in situ* gelation after administration.

**Fig. S12.** Immunohistochemical staining of type I collagen in SKOV3 xenograft tumor tissues. Scale bar: 1000 μm.

**Fig. S13.** Appearance of the antibody-loaded dual-affinity hydrogel.

**Fig. S14.** Aggregation‑caused quenching (ACQ) of Cy5.5‑labeled trastuzumab (TraCy5.5) in the dual‑affinity hydrogel. (A) Representative in vitro IVIS fluorescence images and (B) quantitative analysis of TraCy5.5 in the dual‑affinity hydrogel versus free solution (n = 3). (C) Representative in vivo IVIS fluorescence images and (D) quantitative analysis following subcutaneous injection of TraCy5.5 in the dual‑affinity hydrogel versus free TraCy5.5 (n = 3). In both in vitro and in vivo settings, the hydrogel group exhibited significantly lower fluorescence intensity than the free Tra group, confirming the occurrence of ACQ.

**Fig. S15.** Photographs of excised tumors from each treatment group at the end of the experiment. The saline control group was terminated prematurely on day 22 for ethical reasons (excessive tumor burden and body weight loss), whereas all other groups were maintained until day 28. All tumors were imaged together for direct visual comparison.

**Fig. S16.** H&E staining results of major organs in mice from different formulation groups post-treatment. Scale bar: 100 μm.

**Fig. S17.** H&E staining results of skin tissues at the axillary injection sites in mice administered different formulations. Scale bar: 100 μm.

**Fig. S18.** Analysis results of liver function parameters: (A) alanine aminotransferase (ALT), (B) aspartate aminotransferase (AST), (C) albumin (ALB), (D) alkaline phosphatase (ALP), (E) total protein (TP); and renal function parameters: (F) uric acid (UA), (G) creatinine (CRE), (H) urea in mice from different formulation groups post-treatment.

**Table S1.** Dosage uniformity of different hydrogel formulations upon injection (target volume: 100 μL).

| Parameter | Saline | Tra@Gel | Tra@CBP-Gel | Tra@MEP-Gel | Tra@Dual-affinity Gel |
| --- | --- | --- | --- | --- | --- |
| Expelled mass (mean ± SD, mg) | 100.3 ± 1.2 | 96.2 ± 1.4 | 95.6 ± 1.9 | 95.5 ± 2.3 | 96.4 ± 1.8 |
| CV (%) | 1.2 | 1.5 | 2.0 | 2.4 | 1.9 |

Note: Each formulation was loaded into a 1 mL syringe (26G needle) and manually expelled. The expelled material was weighed (n = 3 per group). The density of all formulations was assumed to be approximately 1 g/mL; thus, the measured mass (mg) directly corresponds to the injection volume (μL). All groups yielded a coefficient of variation (CV) below 3 %, confirming excellent dosage uniformity.
